# Trust Profiles: Associations With Psychological Need Satisfaction, Work Engagement, and Intention to Leave

**DOI:** 10.3389/fpsyg.2021.563542

**Published:** 2021-06-17

**Authors:** Marita Heyns, Sebastiaan Rothmann

**Affiliations:** ^1^North-West University, Vanderbijlpark, South Africa; ^2^Optentia Research Unit, North-West University, Vanderbijlpark, South Africa

**Keywords:** trust, psychological need satisfaction, work engagement, intention to leave, latent profile analyses

## Abstract

The study aimed to identify trust profiles in the work domain and to study how these patterns related to psychological need satisfaction, work engagement, and intentions to leave. A cross-sectional survey with a convenience sample (*N* = 298) was used. The Behavioral Trust Inventory, the Work-related Basic Need Satisfaction Scale, the Work Engagement Scale, and the Turnover Intention Scale were administered. The results showed four trust profiles: skeptic, reliance-based, moderately cautious, and optimistic trustors represented participants' responses on behavioral trust. Skeptic and optimistic trustors (who represented about 50% of the sample) differed primarily regarding their reliance and disclosure intensity. The other two trust profiles (representing the other 50% of the sample) reflected higher reliance and lower disclosure or lower reliance and higher disclosure. Psychological need satisfaction (comprised of autonomy, competence, and relatedness satisfaction) and work engagement were the strongest and intentions to leave the weakest for optimistic trustors (compared to skeptic trustors).

## Introduction

Trust can be a powerful social resource in the workplace if we understand how to unlock its benefits. Trust is vital for effective interpersonal relationships and the achievement of positive workplace outcomes (Colquitt et al., 2007; Costa and Anderson, 2011; Fulmer and Gelfand, 2012; De Jong et al., 2016; Choi and Resick, 2017; Van der Werff and Buckley, 2017). Studies (Dirks and Ferrin, 2002; Schaubroeck et al., 2011; Nienaber et al., 2015; Bligh, 2017; Choi and Resick, 2017) have also shown that trust in higher reports advances followers' work performance. However, due to the intricate nature of this multidimensional, complex construct (De Jong et al., 2016), several basic trust processes still need further clarification (Li, 2007, 2012).

Nienaber et al. (2015) highlight the need to pay more attention to how trust in dyadic workplace relationships unfolds and to focus more on subordinate qualities. They also stress that more heterogeneity—such as employing different methodologies—is needed to advance research in this field. Importantly, and in addition to studying antecedents and outcomes of trust, it is further advised that researchers must appreciate the complex interactions between compound sets of variables underlying the essence of trust. However, studies taking an individual perspective are sparse, and the way trust as the representation of conflicting priorities within an individual unfolds is mostly unknown (Cheng et al., 2017).

Our study offers a unique contribution in that it focuses on a more recent definition of trust where the essence of this construct is operationalized as a decision or actual intent initiated by the trust agent self (Gillespie, 2003, 2012). This option was preferred because it examines the actions of the trusting party as the primary focus of attention, thereby augmenting insights gained by traditional approaches that divert attention to the focal “other” through its emphasis on the trusted party characteristics as primary determinants of trust.

Secondly, we respond to recommendations by Nienaber et al. (2015) to pay more attention to trust within dyadic relationships and specifically by focusing on subordinate qualities, since our study identifies the trust agent as a subordinate in relation to a focal other in a position of authority. Our study further addresses the need to embrace more heterogeneity and dissimilar methodologies in trust research (Nienaber et al., 2015) by employing latent profile analyses (LPA) in an applied workplace setting. By doing so, we also take heed of recommendations by Spurk et al. (2020) that the application of LPA should be encouraged. LPA is a relatively new technique that holds the potential to advance sophisticated theoretical thinking and deepen understanding of the complex, diverse ways in which variables of interest may manifest in applied settings; they further recommend more extensive application of this technique in work and organizational research contexts in particular.

Finally, by combining variable- and person-oriented analyses in the same study, we offer insight into intra-individual trust configurations and describe similar patterns shared among sub-populations. Furthermore, we enhance understanding of how these shared patterns may uniquely relate to the satisfaction of work-relevant basic psychological needs, work engagement, and intentions to leave, which are all critical performance-related success factors.

As far as we are aware, to date, no research has employed a latent profile analysis (LPA) technique to illuminate how trust intentions specifically interact within individuals and how alternative patterns of internal interaction may differentially relate to performance outcomes. We base this on an extensive search employing several search engines, which rendered no relevant results and recent reviews of the literature on LPA (see Woo et al., 2018; Spurk et al., 2020) that did not record any similar studies either.

## The Current Study

The current study adopted a person-centered approach to gain insight into the trust intentions of a trusting party, otherwise known as the trustor, toward a focal person (the trustee) in a position of authority (the direct leader) in a work context. We examined the simultaneous occurrence of the alternative forms of trusting intentions (Gillespie, 2003, 2012)—namely, the willingness to rely on, and disclose to, others—within individuals (trustors) using latent profile analyses.

Variable-oriented or person-oriented approached can be used to study individuals' trust profiles (Collins and Lanza, 2010). The emphasis in a variable-oriented approach (such as factor analysis) is to identify relations between observed variables that apply to all people. In contrast, in person-oriented approaches, the emphasis is on individual patterns relevant to the problem under consideration. Latent profile analysis (LPA) is a type of person-oriented analysis. It uses mixture modeling to identify unobserved subpopulations comprising similar individuals (Wang and Wang, 2020). Mixture modeling offers the opportunity to identify unknown, a priori, distinct profiles of individuals based on the measurement of preferences for reliance and disclosure components, examines the features of unobserved population heterogeneity, and evaluates the effects of covariates on latent profile membership. The study aimed to identify trust profiles in the work domain and study how these patterns related to motivation, work engagement, and turnover intentions.

## A Person-Centered Approach to Trust

Trust research in organizational contexts widely accepts the definition of trust developed by Rousseau et al. (1998) as a point of departure (Nikolova et al., 2015). According to this definition, trust is defined as “a psychological state comprising the intention to accept vulnerability based upon positive expectations of the intentions or behavior of another” (Rousseau et al., 1998, p. 395). However, trust is a complex construct and varying bases used as proxy for trust often leads to confusion and difficulty to compare findings—e.g., researchers might claim that they are studying the trust phenomenon but are actually studying trustworthiness (Cheng et al., 2017).

In their seminal work, Mayer et al. (1995) attempted to provide more clarity by proposing a model that captures the “willingness … to be vulnerable” (p. 712) as the defining element of trust and which should be clearly differentiated from its antecedents (such as propensity and trust beliefs or perceived trustworthiness) and outcomes. This differentiation is important to acknowledge since trustworthiness beliefs are based on qualities that the trusted party possesses, whereas trust itself is something that the trusting party does—although the two concepts are related, they should not be considered as interchangeable alternatives (Mayer et al., 1995; Lam et al., 2013; Cheng et al., 2017). At its core, trust essentially requires a leap of faith (Nikolova et al., 2015; Cheng et al., 2017), as demonstrated by the trusting party's intentional acceptance of vulnerability toward a trusted other without guarantees regarding the other's motives or a favorable outcome (Schoorman et al., 2007; Gillespie, 2012).

Building on the model of Mayer et al. (1995), Gillespie (2003, 2012) proposed operationalization of trust through two conceptually distinct—yet complementary—components, namely, reliance and disclosure. The reliance-based component is defined as a trusting party's willingness to depend on a trustee (Lee et al., 2010) and manifests in a trustor's willingness to accept influence from another person, such as to depend on the trustee's skills and judgement. In contrast, disclosure-based trust is defined as a willingness to disclose either personal or work-relevant information to the trustee (Lee et al., 2010). This could, for example, be expressed in the willingness to communicate openly and honestly and to share personal ideas, opinions, and emotional reactions with a trusted party, even if doing so could be potentially incriminating (Gillespie, 2012).

Gillespie's operationalization of trust diverges from closely related alternative models such as the cognition- and affect-based trust model of McAllister (1995), which focuses on trust beliefs grounded upon cognitive assessments of the trust referent's competence or upon emotional bonds that exist between the trusting parties. In contrast, Gillespie's model focuses explicitly on the actual trust behavior of the trust agent self as the primary focus of interest and is more closely associated with the ideas forwarded by Zand (1972), who identified accepting influence and sharing information as closest proxies for actual trust.

Gillespie's (2012) conceptualization of trust has received substantial support in the literature (Schoorman et al., 2007; Van der Werff and Buckley, 2017), including from McEvily and Tortoriello (2011), who in their review of state-of-the-art trust measures recommended the more extensive use of Gillespie's (2003, 2012) measurement instrument in future studies.

The conceptualization of trust as two different but related dimensions is consistent with the observation that a person can opt to trust in selective ways, leading to widely diverging consequences (Gillespie, 2003; Lam et al., 2013; Cheng et al., 2017). This is in line with research findings, including a meta-analysis on trust-related antecedents and consequences (Dirks and Ferrin, 2002) which showed that different dimensions of trust are not only associated with dissimilar psychological processes but may each contribute to performance outcomes in unique ways (Dirks and Ferrin, 2002; Lee et al., 2010; Lam et al., 2013; De Jong et al., 2016; Cheng et al., 2017; Heyns and Rothmann, 2018).

Gillespie's (2003; 2012) relatively more recent definition of trust has intensified the need to understand how trust decisions develop from an individual perspective and how they relate to other trust-relevant variables (Van der Werff and Buckley, 2017). To illustrate: A recent study by Terblanche and Heyns (2020) showed that neither personality traits nor propensity to trust are predictors of the trust behavior of a trusting party toward a coach within a coaching relationship. Only perceived trustworthiness predicted both reliance and disclosure-based trust in a coach. More specific to this study, a study by Heyns and Rothmann (2018) demonstrated that reliance—which could be understood as a more passive trusting stance whereby one positively accepts influence- did not predict satisfaction of basic motivational needs such as competence, relatedness and autonomy and did not predict work engagement. In contrast, their study found that only disclosure-based trust—which requires a higher degree of personal investment as evidenced in active sharing of different types of information—was a predictor of both basic psychological need satisfaction and of work engagement. Along similar lines, Lam et al. (2013) also found that reliance-based trust had no e?ect on extra-role performance, whereas disclosure-based trust did positively impact.

Wang and Hanges (2011) pointed out that organizational researchers are often familiar with variable-centered approaches (e.g., factor analysis) to understand individual and organizational behavior. Variable-centered analyses capture the interrelatedness among variables to theorize underlying processes. Previous research on trust has relied on a variable-centered approach which assumes that participants are drawn from a single population. Person-centered approaches such as latent profile analyses, on the other hand, seek to identify subpopulations in a sample (Meyer and Morin, 2016). Such analyses focus on the interrelatedness among variables as a function of the heterogeneity of the population. Using latent profile analysis (LPA) may help organizational researchers model phenomena more accurately and realistically (Wang and Hanges, 2011). The value of LPA is that it could capture unobserved heterogeneity of measurement functioning. Variable-centered analyses fail to consider the possible combined effects of different trust dimensions (Caesens et al., 2020). Therefore, person-centered analyses are valuable to explore inconsistent or unexpected variable combinations.

When examining theoretical frameworks that contain dynamic and interactionistic within-person conceptualisations (Rouse et al., 2020), for our purpose, the person-centered approach is preferable to traditional variable-centered approaches because it recognizes that variables may not necessarily combine in identical ways for all types of individuals (Meyer et al., 2015; Meyer and Morin, 2016). Little is known about the combinations of trust dimensions and the associations thereof with psychological well-being, work engagement and intention to leave of employees. The person-centered approach toward analyses in this study can address the following questions: a) Can we identify different trust profiles characterized by matching levels of trust across dimensions, and profiles characterized by different levels of trust among dimensions? b) Is a profile with high levels on reliance and disclosure trust items associated with more positive outcomes (and less negative outcomes) than a profile with other combinations of reliance and disclosure items? LPA can reveal how various profiles relate to differences in behavioral and other performance-related outcomes because it can capture a more significant number of complex simultaneous interactions between variables more clearly and still render interpretable results (Meyer et al., 2015).

It is the first study of which we are aware that highlights trust configurations within individuals. Our study, therefore, provides a nuanced understanding of trust intentions by identifying patterns in which reliance and disclosure intentions interact within individuals in a population that may otherwise not have been evident. Our research is also the first to record which trust profiles are the most prominent and show how diverse profiles relate to psychological need satisfaction, work engagement, and intention to leave. Because LPA enables us to study more complex interactions between variables simultaneously, we can contribute new insights to the existing body of research literature, which might further help to clarify perceived inconsistencies in prior research. At a practical level, insights obtained through LPA are likely to yield more meaningful and cost-efficient interventions directly targeted at relevant subpopulations of workers (Howard and Hoffman, 2017). More in-depth insight into the trust profiles and their associations with psychological need satisfaction, work engagement, and intentions to leave can lead to more closely tailored and cost-effective trust interventions.

## Psychological Need Satisfaction, Work Engagement, and Intention to Leave

Trust in a leader is highlighted as the most powerful factor that influences employees' workplace attitudes, behaviors and performance outcomes and further research is therefore encouraged to understand its impact on work outcomes more fully (Bligh, 2017; Cheng et al., 2017). For instance, leader trust provides a critical motivational foundation that inspires workers to meet and even exceed expectations (Bligh, 2017; Cheng et al., 2017). Research has also shown that trust in a leader is a precursor of employees' intrinsic motivation, which in turn, facilitates important positive outcomes such as work engagement (Shu, 2015). It has a direct buffering effect against negative workplace experiences so that employees who trust their leaders are more likely to stay focused and engaged in their work and less inclined to resign (Bligh, 2017). What is not yet clear is how internal considerations might dynamically interact to demonstrate trust in pertinent ways and how these might further affect the mentioned outcomes in potentially diverging ways.

Basic psychological needs theory (BPNS) proposes that the satisfaction of three universal psychological needs—the need for autonomy, relatedness, and competence—is essential for adjustment, growth, and optimal functioning of individuals (Ryan and Deci, 2017). Autonomy cannot be equated to independence but rather signifies a sense of volition and authenticity as expressed in choice and ownership of tasks (Ryan and Deci, 2017), which may include a willing dependency on others for inputs or direction when needed (Vansteenkiste et al., 2020). Competence concerns the desire to feel capable of achieving valued outcomes. Furthermore, relatedness represents the need to develop close relationships with significant others (Deci and Ryan, 2000; Ryan and Deci, 2017; Rouse et al., 2020) and to feel connected to something bigger than oneself (Ryan and Deci, 2017).

Research shows that psychological need satisfaction is essential to promote and maintain intrinsic motivation (Ryan and Deci, 2017; Vansteenkiste et al., 2020). However, the satisfaction of each need may not necessarily have identical consequences (Deci and Ryan, 2000). Furthermore, each of the three needs uniquely and interactive ways contributes to well-being irrespective of age, milieu, or culture (Vansteenkiste et al., 2020). Although no previous studies could be found that employed LPA in relation to the topic of interest, one study was identified that showed that only disclosure-based trust predicted satisfaction of autonomy needs, which, in turn, mediated the effect of trust on work outcomes (Heyns and Rothmann, 2018).

Work engagement refers to a cognitive-affective state of mind that is of a positive, fulfilling, pervasive, and persistent nature that is recognized by the vigor, absorption, and dedication invested in one's work role (Schaufeli et al., 2002, 2019). Vigor implies high effort, energetic investment, and resilience. Dedication relates to a sense of inspiration, enthusiasm, challenge, pride, and significance. Absorption refers to a flow-like experience where one is immersed in deep concentration, accompanied by a sense that time is passing very quickly, and where one experiences difficulties with detaching oneself from one's work (Schaufeli et al., 2002, 2019). A study by Heyns and Rothmann (2018) showed that reliance-based trust did not significantly influence engagement; only disclosure-based trust predicted work engagement.

Intention to leave signals an attitudinal orientation toward leaving an organization and is a reliable indicator of subsequent turnover (Costigan et al., 2012; Chan and Mai, 2015). Previous research has indicated that contextual factors affect intentions to leave. Cognition-based trust in a leader is inversely related to employee turnover, while emotion-based trust exhibits a curvilinear relationship to employee turnover (Costigan et al., 2012).

## Method

### Participants

A convenience sample targeting participants from a South African agricultural business with a qualification of Grade 12 or higher (*N* = 298) was used. Agricultural businesses are an important sector of the South African economy. These businesses provide food for consumption, employ people, and contribute significantly to foreign exchange (Meijerink and Roza, 2007; Venter, 2017). Most of the respondents were white (58.3%) males (46.7%), which corresponded well to the company's overall demographic profile. Most participants had Grade 12 as their highest qualification (39.3%) or had either a diploma or a degree (37.9%). The job levels ranged from non-managerial (32%) to supervisory (36.5%), middle-level management (23.4%), and senior management (0.8%). A total of 7% of participants were unsure of their job levels.

### Measuring Instruments

Four previously validated measuring instruments, namely the Behavioral Trust Inventory (BTI; Gillespie, 2003), the Work-related Basic Need Satisfaction Scale (WBNSS; Van den Broeck et al., 2010), the Work Engagement Scale (WES; May et al., 2004), and the Turnover Intention Scale (TIS) (Sjöberg and Sverke, 2000) were used in the study.

Gillespie (2003) devised the BTI to assess a person's trust behavior within a relationship with a specified focal person, and it significantly contributes to predicting key leadership outcomes to a better extent than alternative measures of trustworthiness (Gillespie, 2012). The BTI is a 10-item measurement instrument of reliance-based trust (five items) and disclosure-based trust (five items). The correlation between the reliance-based and disclosure-based trust was 0.67 in this study. Participants rate their willingness to demonstrate trusting behaviors toward their direct higher reports (leaders) on a seven-point rating scale ranging from 1 (*not at all*) to 7 (*completely*). An example item on the reliance scale is “How willing are you to depend on your leader to back you up in difficult situations?”. The disclosure scale measures trust at a more personal level (Lee et al., 2010). An example item is “How willing are you to share your personal beliefs with your leader?” Previous research has reported Cronbach's alpha values well above 0.80 for both scales (Gillespie, 2003; Lee et al., 2010; Lam et al., 2013).

Three subscales of the WBNSS (Van den Broeck et al., 2010) were used to measure autonomy, competence, and relatedness satisfaction. Six items were used to tap into the respondent's personal experiences at work in terms of the need for autonomy. Each item offers options ranging from 1 (*totally disagree*) to 5 (*totally agree*). An example of an item is “I feel free to do my job the way I think it could best be done.” A study in the South African workplace context confirmed that the scale had a stable three-factor structure and acceptable alpha coefficient of 0.81 (Rothmann et al., 2013).

The WES (May et al., 2004) was used to measure engagement. Based on Kahn's^1990^ conceptualization of engagement, it employs 12 items that measure cognitive elements (Items 1–4; for example, “I get so into my job that I lose track of time,” Item 1), emotional elements (Items 5–8; for example, “I am passionate about my job,” Item 5), and physical elements (Items 9–12; for example, “I am full of energy in my work,” Item 9) of engagement on a frequency scale varying from 1 (*almost never or never*) to 7 (*always or almost always*). In a South African context, Rothmann et al. (2013) found each component to have the following alpha coefficients: physical = 0.80; emotional = 0.82; and cognitive = 0.78.

Employees' intention to leave was measured using the TIS (Sjöberg and Sverke, 2000). This three-item scale measures the strength of the respondent's intention to resign from his/her present position on a five-point scale, ranging from 1 (*totally disagree*) to 5 (*totally agree*); a high score reflects a strong intention to leave. During initial standardization of this scale, an acceptable Cronbach's alpha coefficient of 0.83 was obtained (Sjöberg and Sverke, 2000).

### Research Procedure

The Ethics Committee of the Vanderbijlpark Campus of the North-West University, South Africa, cleared the study (Ethics Approval Number: NWU-00014-14-A8). The management of the target organization also gave permission to administer the questionnaire in the company. Participants received a self-addressed envelope containing a cover letter and a hard copy of the questionnaire. The cover letter explained the purpose of the survey and emphasized that participation was entirely voluntary. Confidentiality and anonymity were guaranteed. After completing the questionnaires, respondents could either return the sealed envelopes to identified employees in the human resource management department or mail them directly to the researcher. The raw data were converted to an SPSS dataset for use in Mplus 8.4.

### Statistical Analysis

Latent profile analysis (LPA) in Mplus 8.4 (Muthén and Muthén, 1998–2020) was used to determine the different trust profiles that fit the data. A stepwise, iterative model comparison process was employed to test a series of models with an increasing number of latent profiles to determine the number of profiles present in the data (Nylund et al., 2007; Geiser, 2010; Wang and Wang, 2020). We retained a model when there was a significant improvement from the reference model to this model with more profiles. The models were evaluated according to the Bayesian information criterion (BIC), Akaike information criterion (AIC), sample-size adjusted BIC (ABIC) values, entropy, and latent class assignment probabilities, comparing the different models. The Lo-Mendell-Rubin (LMR LR) test (Lo et al., 2001), the adjusted LMR LR (ALMR) test, and the bootstrapped likelihood ratio test (BLRT) (Wang and Wang, 2020) were used to test the number of classes in a mixture analysis.

Measurement models of psychological need satisfaction, work engagement and intention to leave were tested using confirmatory factor analysis (CFA) in Mplus 8.4 (Muthén and Muthén, 1998–2020). The weighted least squares mean and variance adjusted (WLSMV) estimator was used. The following fit indices were utilized to assess model fit: the chi-square statistic (the test of absolute fit of the model), Akaike information criterion (AIC) and Bayesian information criterion (BIC), standardized root mean residual (SRMR), root mean square error of approximation (RMSEA), Tucker-Lewis index (TLI), and Comparative fit index (CFI) (West et al., 2012). We used the procedure developed by Saris et al. (2009) to search for misspecifications in models. Saris et al. (2009) combined modification indices with the power of the test to identify misspecifications of models. We used the JRule software package (van der Veld et al., 2008) as adapted for Mplus (Oberski, 2014), which follows the procedures as suggested by Saris et al. (2009).

SPSS 26.0 (IBM Corp., 2020) was used to compute descriptive statistics, and Pearson correlation coefficients were employed to identify the relationships between the variables. Point estimates of scale reliability were computed using confirmatory factor analysis (see Raykov, 2009). A cut-off value for scale reliability of 0.70 (Raykov, 2009) was used.

As a means of determining the mean of a distal continuous outcome across latent profiles, the automatic Bolck, Croon, and Hagenaars (BCH) method was used (Asparouhov and Muthén, 2014; Bakk and Vermunt, 2016).

## Results

### Latent Profile Analysis

As mentioned, a latent profile analysis (LPA) with Mplus 8.4 (Muthén and Muthén, 1998–2020) was carried out based on their responses to the 10 items of the BTI. The fit indices are reported in Table 1.

**Table 1:** Comparison of different latent profile analysis models.

**Model**	**AIC**	**BIC**	**ABIC**	**LMR LR test *p*-value**	**ALMR LR test *p*-value**	**BLRT *p*-value**
1-class LPA	12104.64	12178.58	12115.15	n/a	n/a	n/a
2-class LPA	11158.01	11272.62	11174.31	0.0036^**^	0.0039^**^	0.0000^**^
3-class LPA	10759.26	10914.53	10781.34	0.0258	0.0272	0.0000^**^
4-class LPA	10479.87	10675.81	10507.73	0.0254	0.0265	0.0000^**^
5-class LPA	10376.70	10613.31	10410.35	0.6076	0.6114	0.0000^**^

*AIC, Akaike information criterion; BIC, Bayesian information criterion; ABIC, adjusted Bayesian information criterion; LMR LR, Lo-Mendell-Rubin test; ALMR LR, adjusted Lo-Mendell-Rubin test; BLRT, bootstrapped likelihood ratio test*;

** *p < 0.01*.

As the number of latent profiles is unknown and cannot be directly estimated from the model, various models with different numbers of latent classes were tested, starting with a single profile model and increasing the number of profiles by one each time (Wang and Wang, 2020). In total, five competing models, each containing a different number of profiles, were tested. As is evident from Table 1, the results pointed to Profile 4 as the preferred model. The fit indices showed a significantly better fit for Profile 4 compared with the other profiles. Profile 3 (ΔAIC = 279.39; ΔBIC = 238.72; ΔABIC = 273.61). The LMR LR (*p* > 0.05) and ALMR (*p* > 0.05) were not statistically significant for Profile 4; however, the BLRT (*p* < 0.01) for Profile 4 was statistically significant. Regarding the fit of the five-profile model, the fit indices showed a better fit compared with Profile 4 (ΔAIC = 103.17; ΔBIC = 62.50; ΔABIC = 97.38). The BLRT (*p* < 0.01) for Profile 5 was statistically significant. However, we preferred the 4-profile model because it was interpretable and because the five-profile model had only a small number of participants in one profile.

**Figure 1:**
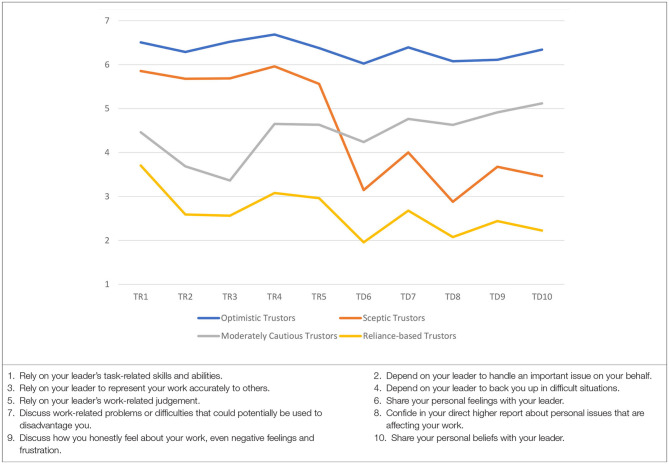
Four latent profiles based on 10 items of the BTI.

The quality of the latent profile relationship was investigated using entropy values. The entropy value was 0.91, indicating a good classification (Wang and Wang, 2020). The average latent class assignment probabilities for individuals assigned to each profile were 0.98 (Profile 1), 0.95 (Profile 2), 0.92 (Profile 3), and 0.97 (Profile 4). Therefore, individuals, on average, were classified with high certainty into their most likely latent profile. Profiles were labeled based on their means for the 10 items of the BTI. The four latent profiles are illustrated in Figure 1.

Profile 1 (skeptic trustors; 14.43% of the sample) had the lowest mean scores. Profile 2 (reliance trustors; 26.17%) had higher scores on Items 1 to 5 of the BTI and lower scores on Items 6 to 10. Profile 3 (disclosure trustors; 23.49%) showed scores of 3.5 and higher on all the items of the BTI. Lastly, Profile 4 (optimistic trustors; 35.19%) obtained high mean scores on all the items of the BTI. The characteristics of the four trust profiles are discussed next.

- **Profile 1—skeptic trustors (14.43%)**. Individuals in Profile 1 were unwilling to rely on their leaders in work-related matters. They were also unwilling to share their personal opinions and emotional experiences with their leaders.
- **Profile 2—reliance-based trustors (26.17%)**. Individuals in Profile 2 were willing to rely on their leaders in work-related matters. However, they were unwilling to share their personal opinions and emotional experiences with their leaders.
- **Profile 3—moderately cautious trustors (23.49%)**. Individuals in profile 3 were willing to accept influence from their leaders within moderate limits only. They were slightly more inclined to share their personal opinions and emotional experiences, although this remained to a modest degree as well.
- **Profile 4—optimistic trustors (35.91%)**. Individuals in Profile 1 were willing to rely on their leaders in work-related matters. They were also willing to share their personal opinions and emotional experiences with their leaders.

When comparing the four profiles, the skeptic trustors found it the most problematic to accept vulnerability toward a person in a position of authority. In contrast, the optimistic trustors found it the easiest to do so. A closer examination of preferences regarding either reliance- or disclosure-based trust intentions revealed that the optimistic trustors were equally comfortable relying on, and disclosing to, their leaders with ease. Both the skeptic and reliance-based trustors preferred to express trust intentions in terms of reliance, although they differed in the extent to which they were willing to do so. The moderately cautious trustors differed from all the other groups in the sense that they were more inclined to demonstrate trust through the expression of their personal opinions and feelings rather than through reliance-based acceptance of vulnerability toward leaders in situations over which they had no or little control.

### Association Between Latent Profiles, Psychological Need Satisfaction, Work Engagement, and Intention to Leave

Next, the associations between the four latent profiles and psychological need satisfaction, work engagement, and intention to leave as auxiliary variables are reported.

#### Testing the Measurement Model of Distal Variables

Based on the results of previous studies (Rothmann et al., 2013; Rothmann and Fouché, 2018) regarding the factor structures of the distal variables included in this study, we decided to test one measurement model. The model consisted of five latent variables: autonomy satisfaction (measured by six items), competence satisfaction (measured by six items), relatedness satisfaction (measured by six items), work engagement (measured by 12 items), and intention to leave (measured by three items).

The following fit statistics were obtained for this measurement model: χ^2^ = 2,168.48 (*df* = 485, *p* < 0.0001), RMSEA = 0.11 [0.103, 0.113], *p* < 0.0001, CFI = 0.89, TLI = 0.88, SRMR = 0.09. None of the fit indices was acceptable, so respecification of the model was necessary. In line with the recommendations of Saris et al. (2009), we used the JRule software package to detect misspecifications in the model using modification indices (MI), expected parameter change (EPC), and the power of the MI test. While one misspecification might invalidate a model, it has been argued that models typically have various misspecifications because they simplify reality (MacCallum et al., 1996; Lukat et al., 2016). Accordingly, we aimed for factor models that were adequate from a practical perspective, in other words, when misspecifications of parameters did not change the interpretation of the model (see Lukat et al., 2016). Using the guidelines of serious model misspecification of Saris et al. (2009), namely that MI is significant, the power of the MI test is high, and the EPC is large (>0.20), we detected three problematic items, namely item 4 of relatedness satisfaction (“At work, I can talk with people about things that really matter to me”), item 3 of autonomy satisfaction (“If I could choose, I would do things at work differently”), and item 4 of work engagement (“When I'm working, I often lose track of time”).

The respecified measurement model showed acceptable fit statistics: χ^2^ = 1,156.04 (*df* = 395, *p* < 0.0001), RMSEA = 0.07 [0.07, 0.08], *p* < 0.0001, CFI = 0.95, TLI = 0.94, SRMR = 0.07. The model misspecification results showed that the remaining parameters were not misspecified (i.e., insignificant MIs and high power), were inconclusive (low MIs and low power), or they had significant MI and high power, but low EPCs (<0.20).

#### Descriptive Statistics, Reliabilities, and Correlations

The descriptive statistics, reliabilities, and Pearson correlations of the distal variables are reported in Table 2.

**Table 2:** Descriptive statistics, reliabilities, and Pearson correlations of the distal variables.

**Variable**	**ρ**	**Min**.	**Max**.	**Mean**	***SD***	**1**	**2**	**3**	**4**
1. Autonomy satisfaction	0.75	−1.27	1.44	−0.01	0.55	-	-	-	-
2. Competence satisfaction	0.89	−1.26	1.38	0.00	0.58	0.68^*^	-	-	-
3. Relatedness satisfaction	0.80	−0.91	1.01	−0.01	0.42	0.67^*^	0.63^*^	-	-
4. Work engagement	0.94	−1.33	0.86	−0.00	0.38	0.72^*^	0.47^*^	0.33^*^	-
5. Intention to leave	0.90	−1.08	1.90	0.05	0.62	−0.66^*^	−0.22^*^	−0.42^*^	−0.41^*^

* *p < 0.01*.

Table 2 shows that acceptable reliability coefficients higher than 0.70 (Raykov, 2009) were obtained for the five scales that measured autonomy, competence, and relatedness satisfaction, work engagement, and intention to leave. The correlations in Table 2 show that autonomy satisfaction is strongly and positively related to competence satisfaction, relatedness satisfaction, and work engagement and negatively related to intention to leave. Competence satisfaction is strongly and positively related to relatedness satisfaction and moderately and negatively related to intention to leave. Relatedness satisfaction is moderately positively related to work engagement and negatively related to intention to leave. Furthermore, work engagement is moderately and negatively related to intention to leave.

#### Latent Profiles and Distal Outcomes

The differences between the results of the BCH approach are reported in Table 3.

**Table 3:** Equality tests of means across profiles.

**Autonomy satisfaction**			**Competence satisfaction**		
	**Mean**	**SE**		**Mean**	**SE**
Profile 1	−0.37	0.07	Profile 1	−0.28	0.07
Profile 2	−0.21	0.06	Profile 2	−0.15	0.06
Profile 3	0.01	0.07	Profile 3	0.04	0.08
Profile 4	0.28	0.05	Profile 4	0.21	0.06
**Chi-square tests**	***χ***^**2**^	***p***		***χ***^**2**^	***p***
Overall test	70.85	0.00^**^	Overall test	33.60	0.00^**^
Profile 1 vs.2	2.95	0.09	Profile 1 vs.2	1.97	0.16
Profile 1 vs.3	15.20	0.00^**^	Profile 1 vs.3	8.66	0.00^**^
Profile 1 vs.4	56.99	0.00^**^	Profile 1 vs.4	28.46	0.00^**^
Profile 2 vs.3	5.90	0.02^*^	Profile 2 vs.3	3.06	0.08
Profile 2 vs.4	39.74	0.00^**^	Profile 2 vs.4	17.08	0.00^**^
Profile 3 vs.4	10.40	0.00^**^	Profile 3 vs.4	3.04	0.08
**Relatedness satisfaction**			**Work engagement**		
	**Mean**	**SE**		**Mean**	**SE**
Profile 1	−0.20	0.05	Profile 1	−0.15	0.05
Profile 2	−0.13	0.04	Profile 2	−0.14	0.04
Profile 3	0.06	0.05	Profile 3	−0.01	0.04
Profile 4	0.13	0.05	Profile 4	0.17	0.04
**Chi-square tests**	***χ***^**2**^	***p***		***χ***^**2**^	***p***
Overall test	31.63	0.00^**^	Overall test	41.34	0.00^**^
Profile 1 vs. 2	1.04	0.31	Profile 1 vs. 2	0.02	0.90
Profile 1 vs. 3	12.38	0.00^**^	Profile 1 vs. 3	4.24	0.04
Profile 1 vs. 4	24.18	0.00^**^	Profile 1 vs. 4	25.02	0.00^**^
Profile 2 vs. 3	7.23	0.01^**^	Profile 2 vs. 3	4.46	0.04^*^
Profile 2 vs. 4	17.03	0.00^**^	Profile 2 vs. 4	30.12	0.00^**^
Profile 3 vs. 4	0.99	0.32	Profile 3 vs. 4	9.62	0.00^**^
**Intention to leave**					
	**Mean**	**SE**			
Profile 1	0.42	0.11			
Profile 2	0.21	0.06			
Profile 3	0.01	0.07			
Profile 4	−0.21	0.06			
**Chi-square tests**	***χ***^**2**^	***p***			
Overall test	37.38	0.00^**^			
Profile 1 vs. 2	2.72	0.10			
Profile 1 vs. 3	9.27	0.00^**^			
Profile 1 vs. 4	24.85	0.00^**^			
Profile 2 vs. 3	4.07	0.04^*^			
Profile 2 vs. 4	23.67	0.00^**^			
Profile 3 vs. 4	6.23	0.01^**^			

* *p < 0.05*;

** *p < 0.01*.

*P1, Skeptic; P2, Reliance-based; P3, Moderately Cautious; P4, Optimistic*.

The results in Table 3 show that statistically significant differences exist between the autonomy satisfaction (χ^2^ = 70.85, *p* < 0.01), competence satisfaction (χ^2^ = 33.60, *p* < 0.01), relatedness satisfaction (χ^2^ = 31.63, *p* < 0.01), work engagement (χ^2^ = 41.34, *p* < 0.01), and intention to leave (χ^2^ = 37.38, *p* < 0.01) of different trust profiles.

As far as autonomy satisfaction is concerned, Table 3 shows that statistically significant differences exist between skeptic and optimistic trustors (χ^2^ = 56.90, *p* < 0.01) as well as reliance-based and optimistic trustors (χ^2^ = 39.74, *p* < 0.01). Moderately cautious trustors differ statistically significantly from skeptic trustors (χ^2^ = 15.20, *p* < 0.01) and reliance-based trustors (χ^2^ = 10.40, *p* < 0.01). Moreover, optimistic trustors differ statistically significantly from moderately cautious trustors (χ^2^ = 10.40, *p* < 0.01).

Concerning competence satisfaction, Table 3 shows that statistically significant differences exist between skeptic and optimistic trustors (χ^2^ = 28.46, *p* < 0.01) as well as skeptic and moderately cautious trustors (χ^2^ = 8.66, *p* < 0.01). Reliance-based trustors differ statistically significantly from optimistic trustors (χ^2^ = 17.08, *p* < 0.01).

As far as relatedness satisfaction is concerned, Table 3 shows that statistically significant differences exist between skeptic and optimistic trustors (χ^2^ = 24.18, *p* < 0.01) as well as skeptic and moderately cautious trustors (χ^2^ = 12.18, *p* < 0.01). Moderately cautious trustors differ statistically significantly from reliance-based trustors (χ^2^ = 7.23, *p* < 0.01). Additionally, optimistic trustors differ statistically significantly from reliance-based trustors (χ^2^ = 17.03, *p* < 0.01).

Concerning work engagement, Table 3 shows that statistically significant differences exist between skeptic and optimistic trustors (χ^2^ = 25.02, *p* < 0.01), reliance-based and optimistic trustors (χ^2^ = 30.12, *p* < 0.01), and moderately cautious and optimistic trustors (χ^2^ = 9.62, *p* < 0.01).

As far as intention to leave is concerned, Table 3 shows that statistically significant differences exist between skeptic and moderately cautious trustors (χ^2^ = 9.27, *p* < 0.01) as well as skeptic and optimistic trustors (χ^2^ = 24.85, *p* < 0.01). Optimistic trustors differ statistically significantly from moderately cautious trustors (χ^2^ = 6.23, *p* < 0.01) and reliance-based trustors (χ^2^ = 23.67, *p* < 0.01).

**Figure 2:**
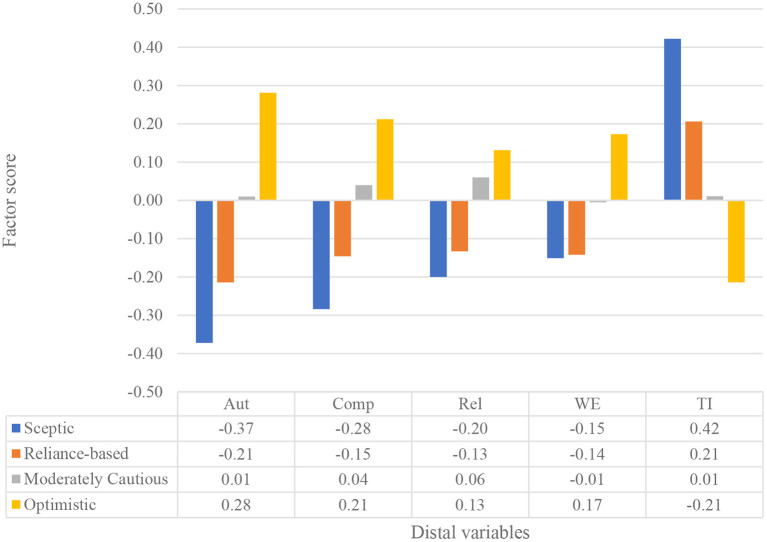
Mean scores of four latent profiles.

The mean factor scores for the four distal variables are presented in Figure 2.

## Discussion

This study aimed to examine the simultaneous occurrence of the alternative forms of trusting intentions within individuals toward a direct leader in a work context using latent profile analyses. We were also interested in seeing how different profiles were associated with psychological need satisfaction, work engagement, and turnover intentions.

Four trust profiles, namely, skeptic trustors, reliance-based trustors, moderately cautious trustors, and optimistic trustors were found in this study. Skeptic trustors were reserved and unwilling to rely on their leaders in work-related matters. They were also unwilling to share their personal opinions and emotional experiences with leaders. Although they were willing to rely on their leaders' task-related skills and abilities, they measured low on almost all the other trust elements of reliance and disclosure. Of all the profiles, skeptic trustors seemed to find it the most difficult to accept vulnerability toward a person in a position of authority.

Reliance-based trustors expressed trust intentions in terms of reliance, but the profile showed a drastic downward tendency for all the disclosure elements. These trustors were not willing to reveal their personal views and feelings to their leaders. While they were prepared to express their opinions and frustrations regarding work-related matters to some extent, they were unwilling to share their beliefs and confide in their leaders about personal issues, even if these might affect their work. In contrast, moderately cautious trustors differed from all the other groups, in that they were more inclined to demonstrate trust through the expression of their personal opinions and feelings. However, they were unwilling to rely on their leaders.

**Figure 3:**
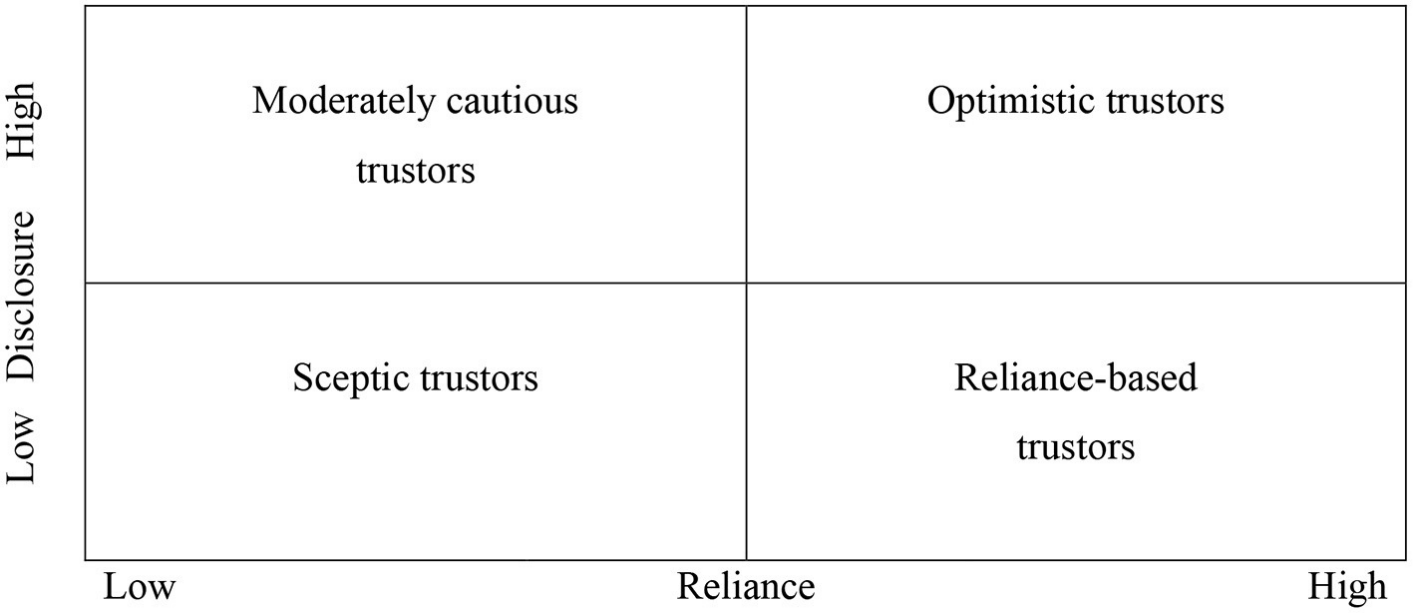
Four trust profiles.

The four types of trustors found in this study are illustrated in Figure 3.

Optimistic trustors experienced significantly higher levels of autonomy satisfaction than the other three profiles and higher levels of competence and relatedness satisfaction than skeptic and reliance-based trustors. Relatively large differences were also found regarding intentions to leave and work engagement of optimistic trustors compared with the other three profiles. Optimistic trustors scored lower on intention to leave and were more engaged in their work than the other types of trustors.

Moderately cautious trustors experienced significantly less autonomy satisfaction, lower work engagement, and higher intentions to leave than optimistic trustors. However, moderately cautious trustors did not differ from optimistic trustors in terms of competence and relatedness satisfaction.

Interestingly, skeptic and reliance-based trustors did not differ significantly regarding psychological need satisfaction, work engagement, and intention to leave levels. However, they showed significantly lower levels of autonomy and relatedness satisfaction, as well as work engagement, and higher intentions to leave than moderately cautious and optimistic trustors. Reliance-based trustors did not differ significantly from moderately cautious trustors concerning competence satisfaction. The findings support and further refine previous claims such as those by De Jong et al. (2016), Dirks and Ferrin (2002), and Schaubroeck et al. (2011) who proposed that cognition-vs. affect-based trust are differentially associated with outcomes.

Findings from previous studies seem to provide perspective regarding the associations between trust profiles and distal variables.

Firstly, employees who show low behavioral trust (in terms of reliance and disclosure) lack a sense of volition and authenticity regarding their tasks (Ryan and Deci, 2017), they feel incapable of achieving valued outcomes, and they do not have close relationships with significant others (Deci and Ryan, 2000; Ryan and Deci, 2017; Rouse et al., 2020). The results showed that low autonomy satisfaction was associated with low reliance and low disclosure. In contrast, high autonomy satisfaction was associated with high reliance and high disclosure. The finding that competence and relatedness satisfaction did not differ significantly between optimistic and moderately cautious trustors suggests that satisfaction of these needs might support the disclosure dimension of behavioral trust. However, moderately cautious trustors showed significantly less autonomy satisfaction than optimistic trustors. The results suggest that autonomy satisfaction is vital for behavioral trust and that relatedness satisfaction supports disclosure-based trust, while competence satisfaction supports reliance-based trust.

Secondly, the findings that optimistic trustors experienced higher work engagement and less intention to leave can be explained by the relational model of work engagement (Kahn and Heaphy, 2014) and the theory of psychological safety (Edmondson, 2019). Psychological safety is linked to the nature of work relationships as the context in which individuals feel free to express themselves, with the vulnerability and exposure that self-expression implies. Such relationships occur in interpersonal relations, inter alia, with leaders. Kahn and Heaphy (2014) refer to the concept of *holding environments* in which people floundering in anxiety are caught up and secured by others (for example, leaders). Such environments have integrity when a sense of safety, created through a series of acts, exists. Kahn and Heaphy (2014) distinguish between three categories of acts a leader might demonstrate, namely, containment (making oneself accessible and receiving others' experiences with compassion and acceptance), empathic acknowledgment (identifying with others as a source of insight), and enabling perspective (helping others make sense of their experiences and orienting others toward their task arrangements). Two mechanisms, namely, identity affirmation (Rosso et al., 2010) and trusting relationships (Schneider et al., 2010), are vital to creating psychological safety (Zhang et al., 2010; Basit, 2017). Relationships that affirm the identity of people create the safety people need to engage at work. Trusting relationships contribute to the emotional carrying capacity needed for employees to feel safe to engage.

In the third place, the results of this study about trust profiles are in line with the findings of various studies. Rothmann and Fouché (2018) linked their findings of intentions to leave to leader-employee relationships (including trust) and psychological need satisfaction. (Kouzes and Posner, 2002, p. 283) related the effects of the leader (and, by implication, trust in the leader) to turnover of employees: “A key factor in why people stay in organizations is their managers. It's equally important in why people leave organizations. People, in fact, don't generally quit companies; they quit managers.”

### Limitations and Recommendations

This study had various limitations. Regarding the scope, the study focused on a specific definition of trust as proposed by Gillespie (2003, 2012), namely, the actual trust intentions of the trusting party. The study did not consider alternative dimensions that are often included in alternative conceptualizations of trust, such as the trustor's assessments of the trustworthiness of the trusted party, but that may also be a potentially influential contextual factor that predicts levels of actual willingness to trust. Therefore, the results should be interpreted with caution, as the assessments of the perceived trustworthiness of the leader may further inform the trust decisions made by followers. Inclusion of trustworthiness as a predictor of trust intentions could be a possible point of departure for future LPA trust studies.

The research design of this study was cross-sectional. Because all the variables were measured simultaneously, it was impossible to prove causal relations between the variables. Moreover, the findings from this study cannot be generalized to other contexts. This study should be replicated with larger samples and in other contexts.

## Conclusion

It can be concluded that a person-centered approach results in a better understanding of behavioral trust and, specifically, how its reliance and disclosure elements combine to form trust profiles. The results showed that four trust profiles, namely, skeptic, reliance-based, disclosure-based, and optimistic trustors, represented participants' responses on behavioral trust well. Skeptic and optimistic trustors (who represented about 50% of the sample) differed primarily regarding their reliance and disclosure intensity. The other two trust profiles (representing the other 50% of the sample) reflected either high reliance and low disclosure or low reliance and high disclosure. Psychological need satisfaction (autonomy, competence, and relatedness satisfaction) and work engagement were the strongest and intentions to leave the weakest for optimistic trustors (compared to skeptic trustors).

The implications of the results of this study for leaders are that command of facts are essential to promote the reliance dimension of trust. In this regard, leaders should demonstrate task-related skills and abilities, demonstrate that they handle important issues for followers well, represent followers' work accurately, back them up in stressful situations, and show good judgement. To promote the disclosure dimension of trust, leaders should demonstrate the emotional capacity to express care and concern when followers share their personal feelings, discuss their work-related and personal problems, and when they share their beliefs.

## Data Availability Statement

The datasets presented in this study can be found in online repositories. The names of the repository/repositories and accession number(s) can be found below: https://www.dropbox.com/s/ht3zrd4ckrtwgzz/data4.sav?dl=0.

## Ethics Statement

The studies involving human participants were reviewed and approved by Human and Social Sciences Ethics Committee. The patients/participants provided their written informed consent to participate in this study.

## Author Contributions

MH developed the literature review. SR did the statistical analyses. MH and SR jointly interpreted and edited the manuscript. All authors contributed to the article and approved the submitted version.

## Conflict of Interest

The authors declare that the research was conducted in the absence of any commercial or financial relationships that could be construed as a potential conflict of interest.
